# Identification and validation of NOLC1 as a potential target for enhancing sensitivity in multidrug resistant non-small cell lung cancer cells

**DOI:** 10.1186/s11658-018-0119-8

**Published:** 2018-11-27

**Authors:** Huaping Huang, Tangying Li, Mingjing Chen, Feng Liu, Haifeng Wu, Jie Wang, Jialiang Chen, Xi Li

**Affiliations:** 1 Department of Respiratory Diseases, The First Affiliated Hospital of Hainan Medical University, Haikou, 570102 Hainan China; 2 Healthcare Department, Hainan General Hospital, Haikou, 570311 Hainan China; 3 Department of Pathology, The First Affiliated Hospital of Hainan Medical University, Haikou, 570102 Hainan China

**Keywords:** Non-small cell lung cancer, Multidrug resistance, Microarray, Differentially expressed genes, A549/MDR, NOLC1

## Abstract

Adjuvant chemotherapy has become the frequently adopted standard therapeutic approach for non-small cell lung cancer (NSCLC). However, the development of multidrug resistance (MDR) is a major obstacle contributing to the failure of chemotherapy. This study aimed to identify genes associated with MDR development that predict tumor response to chemotherapy in NSCLC. In the present study, a multidrug-resistant NSCLC cell sub-line, A549/MDR, was established from the A549/DDP cell line and characterized. The resistance index (RI) of this subline was calculated according to the IC50 of A549/MDR relative to the parental A549/DDP cells. The gene expression profiles of A549/DDP and A549/MDR were obtained using an oligonucleotide microarray (Agilent SureHyb microarray chip). The microarray results were validated by qRT-PCR and selected genes were analyzed by in vitro loss-of-function experiments. Gene expression profiling identified 921 differentially expressed genes (DEGs) according to the selection criteria, in which 541 genes were upregulated and 380 genes were downregulated in A549/MDR compared with A549/DDP cells. We found that these DEGs are involved in diverse biological processes, including ribonucleoprotein complex, drug metabolism, the Hippo signaling pathway and transcriptional misregulation. NOLC1, as one of the identified DEGs, was confirmed to be overexpressed in A549/MDR cells and its knockdown significantly enhanced the drug sensitivity of A549/MDR cells in response to multidrug treatment. Furthermore, knockdown of NOLC1 downregulated the expression levels of drug resistance-associated molecules (LRP and MDR1) in A549/MDR cells. These findings provide a new and comprehensive expression profile of MDR in NSCLC cells. Identification and validation of NOLC1 might be a promising therapeutic strategy for the management of MDR of NSCLC patients.

**Electronic supplementary material:**

The online version of this article (10.1186/s11658-018-0119-8) contains supplementary material, which is available to authorized users.

## Introduction

Lung cancer is the most frequent cause of cancer-related mortality worldwide [1]. As the main clinical type, non-small cell lung cancer (NSCLC) accounts for ~ 85% of all lung cancer with a 5-year survival rate of only ~ 20% [2]. Currently, radical surgery is thought to be the best curative treatment for NSCLC. However, only 10–20% of patients are candidates for surgery at the time of diagnosis and quite a few patients present with advanced stage disease [3]. Adjuvant chemotherapy has become the frequently adopted standard therapeutic approach for NSCLC patients in advanced stages, but the development of multidrug resistance (MDR) reduces its effectiveness and is the crucial factor for the failure of clinical treatment [4–6]. Thus, there is urgent need to further elucidate the molecular mechanisms underlying MDR for better clinical management of advanced NSCLC.

MDR is the phenomenon of simultaneous tumor resistance to several groups of cytotoxic drugs that differ in chemical structure and effects [7], which remains a major clinical obstacle to successful cancer treatment [8]. The mechanisms of MDR have been illustrated as multifactorial and the main mechanisms of MDR are pump and non-pump resistance [9, 10]. Pump resistance is the increased ability of cancer cells to actively efflux drugs, regulated by the ATP-binding cassette (ABC) superfamily of membrane transporters (multidrug resistance protein 1, P-gp/MDR1), ABCC subfamily (multidrug resistance-associated protein, MRP) and ABCG2 [11, 12]. Non-pump resistance is the reduction of apoptotic cancer cells, mediated by Bcl-2, survivin and abnormal signaling transduction [13]. Thus co-delivery of chemotherapeutic agents and drug efflux proteins inhibitor might effectively reverse MDR [14, 15]. Unfortunately, the effectiveness of chemotherapy does not significantly improve in clinical studies in patients [16]. It has become clear that the development of MDR arises not from a single mechanism, but likely from multiple stacked processes. Therefore, the precise molecular mechanism of MDR still needs to be elucidated.

Recently, the advances of microarray technology provide novel insight into a comprehensive analysis of molecular targets involved in MDR to improve the cancer response to chemotherapy [17, 18]. Here we constructed an MDR NSCLC cell line by exposing human A549/DDP to six common drugs used in NSCLC chemotherapy: 5-fluorouracil (5-FU), paclitaxel, mitomycin, vinorelbine tartrate, cisplatin (DDP) and gemcitabine hydrochloride. Then we identified changes in gene expression and pathways associated with the development of MDR in NSCLC. Some of these genes were further validated and their relationships with chemotherapy sensitivity were analyzed in vitro, which will provide us a better understanding of how MDR arises and may help find new approaches for overcoming MDR to improve patient survival.

## Methods

### Reagents and antibodies

Dulbecco’s modified Eagle medium (DMEM), fetal bovine serum (FBS), penicillin (100 U/ml) and streptomycin (100 U/ml) were purchased from Invitrogen (Carlsbad, CA, USA). 5-FU (25 mg/ml), paclitaxel (6 mg/ml), mitomycin (1 mg/ml), vinorelbine tartrate (10 mg/ml), DDP (2 mg/ml), gemcitabine hydrochloride (40 mg/ml) and Cell Counting Kit-8 (CCK-8) were purchased from Sigma Chemical Co. (St. Louis, MO, USA).

### Cell culture and establishment of A549/MDR cells

The DDP resistant A549 cell line (A549/DDP) was obtained from the Institute of Biochemistry and Cell Biology, Shanghai Institutes for Biological Sciences (Shanghai, China). To establish a multidrug resistant cell model (A549/MDR), A549/DDP cells were initially incubated in low concentrations of the above six drugs for 30 min. When stable growth was achieved, A549/DDP cells were constantly exposed to continuously increasing concentrations of the above six drugs every 4–5 weeks for 6 months. All cell lines were cultured in DMEM medium supplemented with 10% FBS, 100 U/ml penicillin and streptomycin in a humidified atmosphere containing 5% CO_2_ at 37 °C. Prior to experimental use, A549/MDR cells were moved to a multidrug-free medium for 2 weeks to eliminate the interference of residual multidrug in the A549/MDR culture system.

### Cytotoxicity CCK-8 assay

CCK-8 assay was performed to assess the drug cytotoxicities of 5-FU, paclitaxel, mitomycin, vinorelbine tartrate, cisplatin, and gemcitabine hydrochloride in vitro. Briefly, A549/DDP and A549/MDR cells were plated in 96-well plates at an initial density of 2 × 10^3^ cells per well. Afterwards, 20 μl of CCK-8 solution was added to each well and incubated for 3–4 h at 37 °C. The absorbance was read at a wavelength of 450 nm using a microplate reader (Bio-Rad Laboratories, CA, USA). The half maximal inhibitory concentration (IC50) was determined via regression analysis between drug concentrations and the cell inhibition rate using SPSS 17.0 software. The ratio of IC50 in A549/MDR to IC50 in A549/DDP was calculated as the resistant index (RI) to further evaluate the cytotoxicity.

### Microarray data analysis

RNA isolation, probe preparation and gene expression microarray were performed according to previously reported protocols [19]. Briefly, total RNAs were isolated from A549/DDP and A549/MDR cells (80–90% confluency) using the Qiagen RNeasy Mini Kit (Valencia, CA, USA) according to the manufacturer’s instructions. Isolated total RNAs were purified using the RNeasy Mini kit (Qiagen, Valencia, CA, USA). Total RNA was labeled using Agilent Quick Amp Labeling and then hybridized to the Agilent SureHyb microarray chip (Agilent Technologies). The microarrays were then automatically washed and scanned using the Agilent DNA Microarray Scanner. The Agilent Feature Extraction software (v11.0.0.1) was used to analyze the scanned image and obtain scaled quantitative information. Data were subsequently normalized and the differentially expressed genes (DEGs) were analyzed using Agilent GeneSpring GX v12.1 software (Agilent Technologies). Genes were regarded as differentially expressed using cut-off criteria of *p*-value < 0.05 and |log FC|-value > 1.5 or < 0.5. All DEGs were further analyzed with unsupervised hierarchical clustering based on the standard correlation of logarithmic transformed data.

### GO and pathway analysis

Functional enrichment analysis was performed using GO (http://www.geneontology.org/). The GO classification characterizes genes based on three domains consisting of cellular component, molecular function and biological process [20]. The DEGs were also utilized in KEGG enrichment analyses to further understand their biological function. Both assays proved statistically significant with *p*-value < 0.05.

### Quantitative real-time PCR (qRT-PCR)

Total RNA was isolated using Trizol Reagent (TAKARA) according to the manufacturer’s protocol. Reverse transcription (RT) of RNA was conducted using a Bestar qPCR RT Kit (DBI) according to the manufacturer’s protocol. The qRT-PCR was performed on the Stratagene Mx3000P real-time PCR system (Agilent) with 20 μl of DBI Bestar SybrGreen qPCR Master Mix. The primers used in this study are listed in Additional file 1: Table S1. The relative gene expression was calculated by the 2^−∆∆ct^ method [21] and normalized relative to GAPDH. Each experiment was done in triplicate.

### Cell transfection

For knockdown of NOLC1, pre-designed siRNA targeting NOLC1 (siNOLC1) and the corresponding negative control (NC) were purchased from RiboBio Co. Ltd. (Guangzhou, China). Subsequently, approximately 4 × 10^5^ A549/MDR cells were plated in a 96-well plate and cultured for 2 days. Then cells were transfected with 50 nM of siNOLC1 or an NC for 48 h using Lipofectamine 2000 (Invitrogen) according to the manufacturer’s instructions and further incubated for 48–72 h.

### Flow cytometric analysis of cell apoptosis

Flow cytometry was performed on the A549/MDR cells using the Annexin V-FITC Kit (ab14085, Abcam, Cambridge, UK). In brief, cells were seeded in six-well plates at a density of 2 × 10^5^ cells/well. After cell transfection, harvested cells were washed twice with PBS and suspended in binding buffer. Then cells were stained with the Annexin V-FITC/PI apoptosis detection kit and the apoptosis rates were measured using a flow cytometer (BD Biosciences, Bedford, MD, USA).

### Hoechst staining assay

Following cell transfection, approximately 3 × 10^5^ A549/MDR cells were plated in six-well plates. After washing twice with PBS, cells were stained with Hoechst 33258 (5 μg/ml; Thermo Fisher Scientific, Inc.) for 20 min in the dark. Then, cells were washed with 0.5% TritonX-100 in PBS and observed under a confocal microscope (LSM800, Zeiss, German). Magnification = 300 X.

### Western blot analysis

Protein was extracted from A549/MDR cells using radioimmunoprecipitation assay (RIPA) buffer (Beyotime, Jiangsu, China). The concentration of protein samples was determined using the bicinchoninic acid (BCA) protein assay kit. Then samples containing 30 μg of total protein were separated by 10% SDS-PAGE and transferred onto PVDF membranes (Roche, Germany). After blocking with 5% non-fat skimmed milk, the membranes were washed, and then incubated with primary antibodies against NOLC1 (1:1000, Abcam), LRP (1:1000, Abcam), MDR1 (1:1000, Abcam), Beclin-1 (1: 1000, Abcam) and GAPDH (1:10000, Abcam) overnight at 4 °C. After washing three times, the membranes were then incubated with HRP conjugated secondary antibodies. Protein bands were visualized using an Enhanced ChemiLuminescence Kit (ECL-Kit, Santa Cruz, USA).

### Statistical analysis

All the in vitro experimental data are shown as means ± standard deviation (SD). All statistical analyses were carried out using the SPSS 19.0 statistical software package (IBM SPSS, Armonk, NY, USA). Differences among different groups were analyzed by either unpaired Student’s *t*-test or one-way ANOVA. *P* values of less than 0.05 were considered statistically significant.

## Results

### Establishment of A549/MDR NSCLC cell line

The A549/DDP cell line was used to create the A549/MDR cell line by exposure to a gradually increasing concentration of multidrug, including 5-FU, paclitaxel, mitomycin, vinorelbine tartrate, DDP and gemcitabine hydrochloride. Approximately 6 months were required to develop A549/MDR cells with stable multidrug resistance. After more than 2 weeks in drug free culture, the cytotoxicity of drugs to the parental A549/DDP and resistant A549/MDR lines was determined by CCK-8 assay. As shown in Table 1, the IC50 values of A549/DDP cells were obviously lower than those of A549/MDR cells under the above six drugs treatments. In addition, A549/MDR cells tolerated a significantly higher concentration of the corresponding inducing drugs compared with A549/DDP cells, as demonstrated by a higher RI value.

**Table 1:** Cytotoxicity of drugs in A549/DDP and A549/MDR cells

Drug	A549/DDP IC50 (μg/ml)	A549/MDR IC50 (μg/ml)	RI
5-fluorouracil	28.17	130.06	4.62
Paclitaxel	4.65	82.82	17.83
Mitomycin	1.43	8.67	6.04
Vinorelbine Tartrate	6.09	12.39	2.04
Cisplatin	5.00	33.51	6.70
Gemcitabine Hydrochloride	1.08	19.01	17.59

*IC50* 50% inhibitory concentration, *RI* resistance index, *DDP* Cisplatin, *MDR* multidrug resistance

### Analysis of gene expression patterns between A549/DDP and A549/MDR

**Fig. 1:**
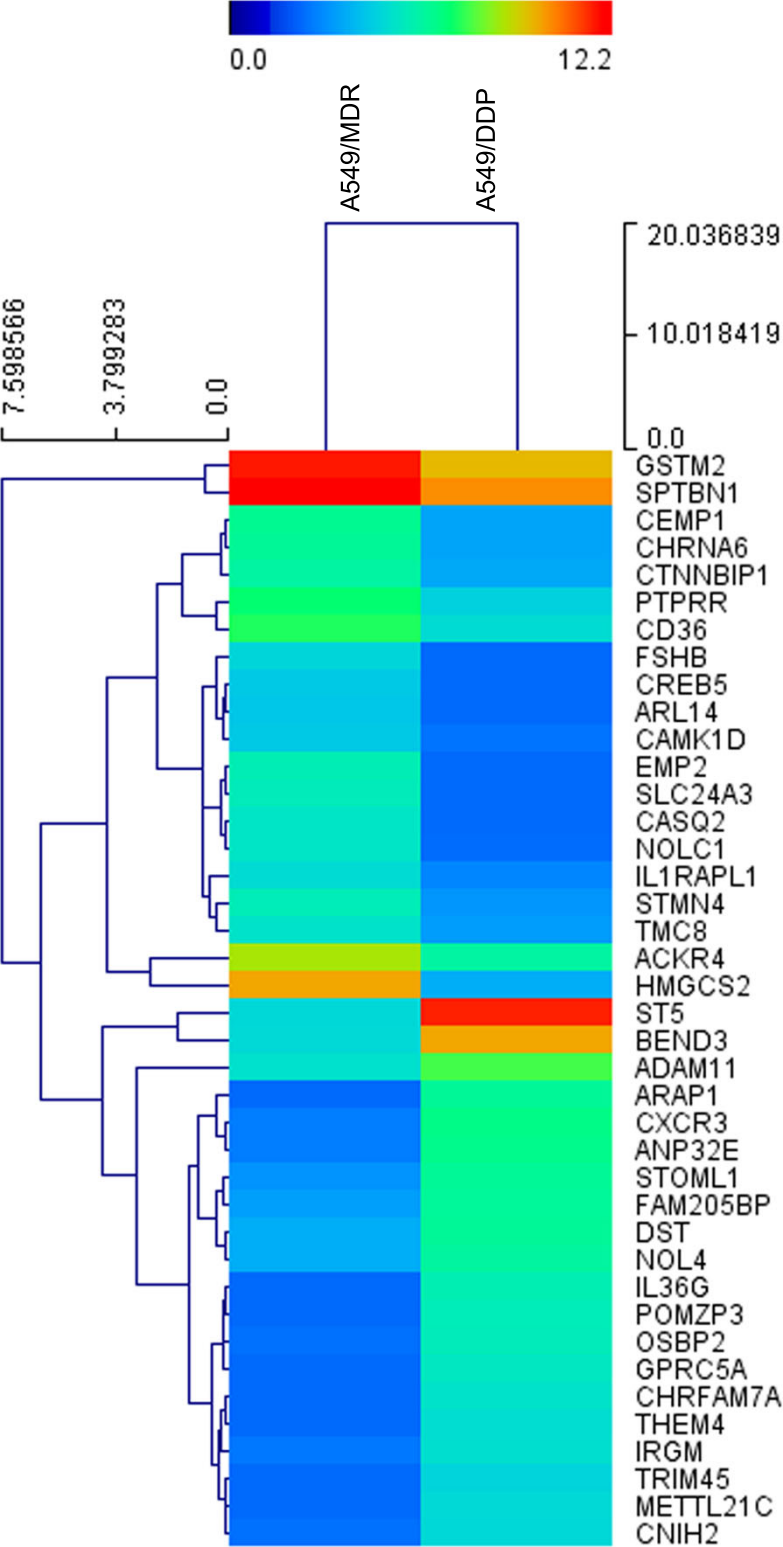
Heat map generated by hierarchical clustering of differentially expressed genes between A549/DDP and A549/MDR cell lines. Rows: genes; Columns: cell lines

To identify potential predictor for chemosensitivity, cDNA microarray was used to analyze the gene expression profiling of the A549/DDP and A549/MDR. The *p*-value < 0.05 and |log FC|-value > 1.5 or < 0.5 were chosen as cut-off criteria. Hierarchical clustering was performed on the two arrays based on the DEGs (Fig. 1). In total 921 DEGs were obtained. Compared to A549/DDP cells, 541 were upregulated and 380 downregulated in A549/MDR cells. The most significant 40 DEGs are presented in Additional file 2: Table S2.

### Functional and pathway enrichment analyses

**Fig. 2:**
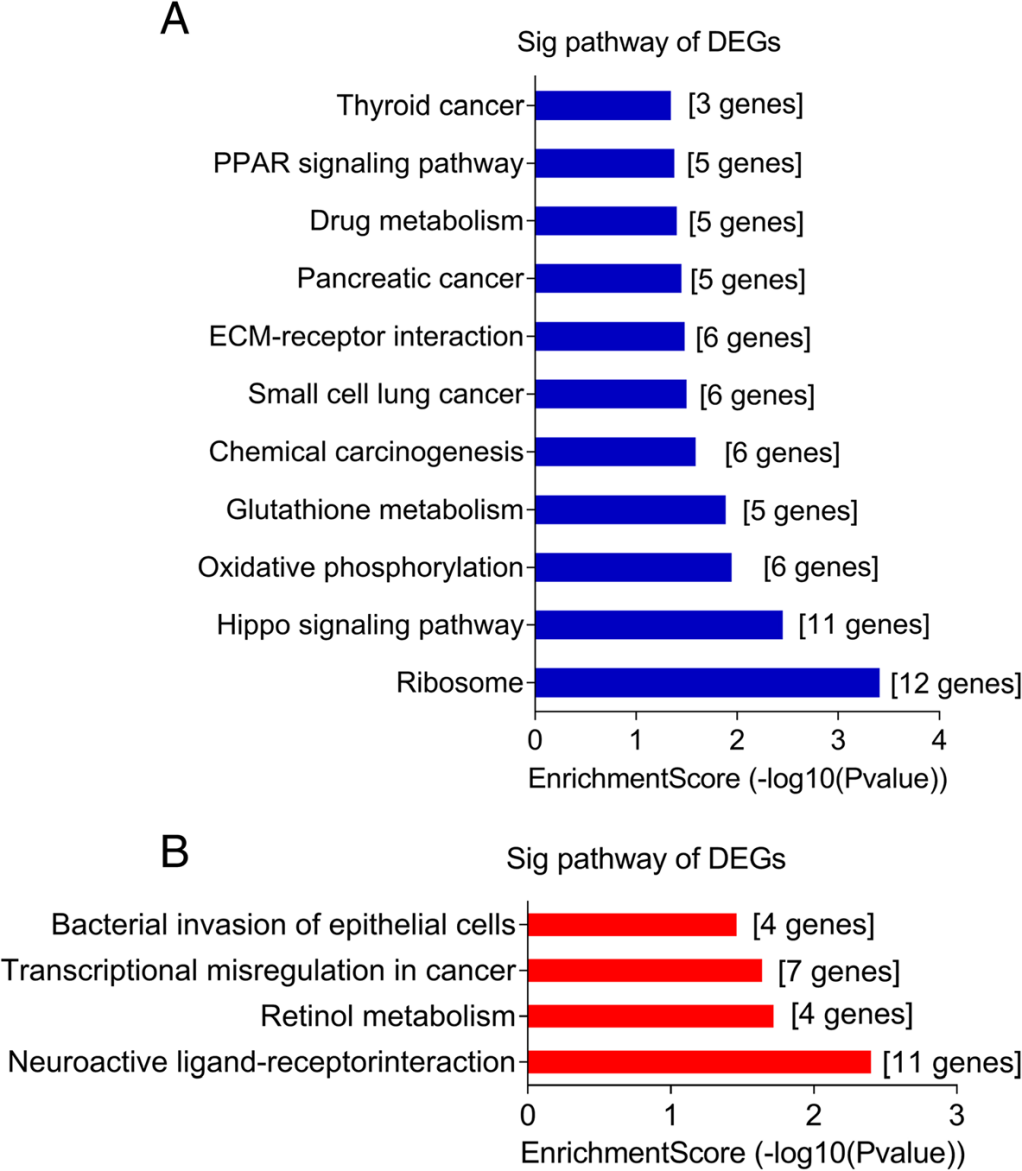
KEGG pathway analyses of differentially expressed genes (DEGs). **a** For upregulated DEGs (the top 11 enriched pathways are presented); and **b** for downregulated DEGs (the top 4 enriched pathways are presented)

GO functional and KEGG pathway enrichment analyses were performed on the aforementioned potential DEGs. The enriched GO functions with a *p* value < 0.05 for the upregulated and downregulated DEGs are presented in Additional files 3 and 4: Tables S3 and Table S4, including the protein localization to membrane and dorsal/ventral pattern formation in the BP category; ribonucleoprotein complex and Cul3-RING ubiquitin ligase complex in the CC category; and structural molecule activity and mRNA 3′-UTR binding in the molecular function MF category. Furthermore, the enriched KEGG pathways for target up- and down-regulated DEGs were analyzed and summarized in Fig. 2a and b, respectively. Genes involved in drug metabolism, chemical carcinogenesis, the Hippo signaling pathway and transcriptional misregulation in cancer might play possible roles in MDR development.

### PCR validation of microarray data

**Fig. 3:**
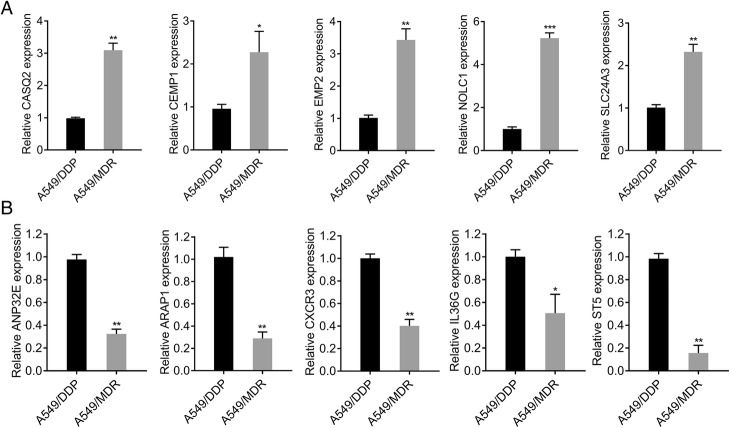
Quantitative real-time PCR was used to confirm the microarray results in A549/DDP and A549/MDR lines. The expression levels of upregulated DEGs (**a**) and downregulated DEGs (**b**) were determined by qRT-PCR in A549/DDP and A549/MDR cell lines. GAPDH was used as an internal control. ^*^p < 0.05, ^**^p < 0.01, ^***^p < 0.001 versus A549/DDP

In total, ten DEGs, including five up- and down-regulated, were screened to verify that the microarray data accurately reflected mRNA levels using qRT-PCR analysis. Consistent with the results of microarray data, the expression levels of CASQ2, CEMP1, EMP2, NOLC1 and SLC24A3 were significantly elevated in A549/MDR cells compared with those in A549/DDP cells (Fig. 3a). Similarly, the expression levels of ANP32E, ARAP1, CXCR3, IL36G and ST5 were remarkably down-regulated in A549/MDR cells in comparison with A549/DDP cells (Fig. 3b). Among these ten verified DEGs, NOLC1 was the most up-regulated and thus was selected for the following experiments. The results showed a good correlation between the microarray and PCR data.

### Knockdown of NOLC1 enhanced the drug sensitivity of A549/MDR cells in response to multidrug

**Fig. 4:**
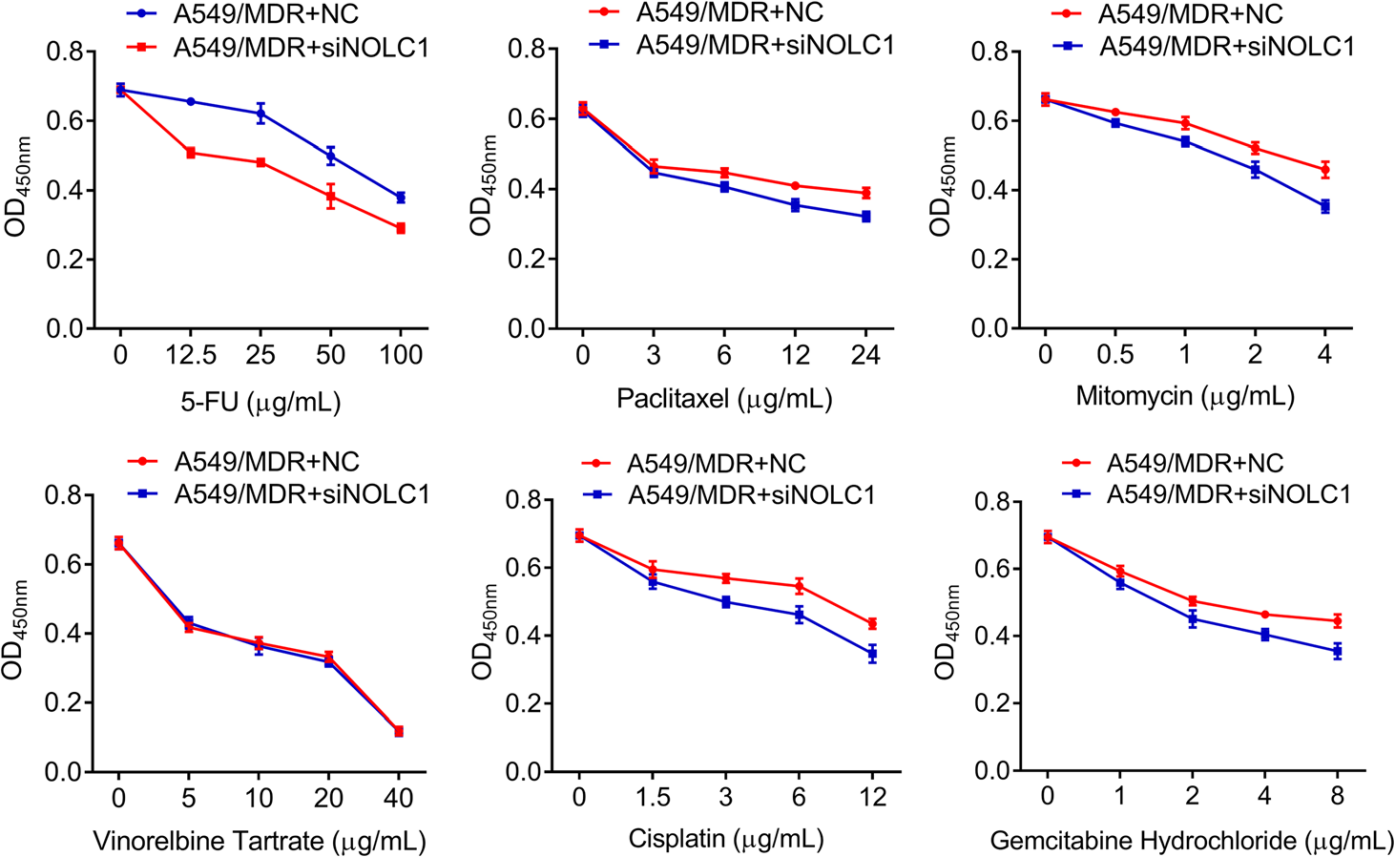
NOLC1 knockdown significantly decreased cell viability of A549/MDR cells after multidrug treatments. A549/MDR cells were transfected with siNOLC1 or NC for 48 h after treatment with 5-FU, paclitaxel, mitomycin, vinorelbine tartrate, DDP and gemcitabine hydrochloride. CCK-8 assay was used to assess the drug cytotoxicities in A549/MDR cells. Data were expressed as means ± SD of at least three experiments. ^*^p < 0.05, ^**^p < 0.01 versus A549/MDR + NC

**Fig. 5:**
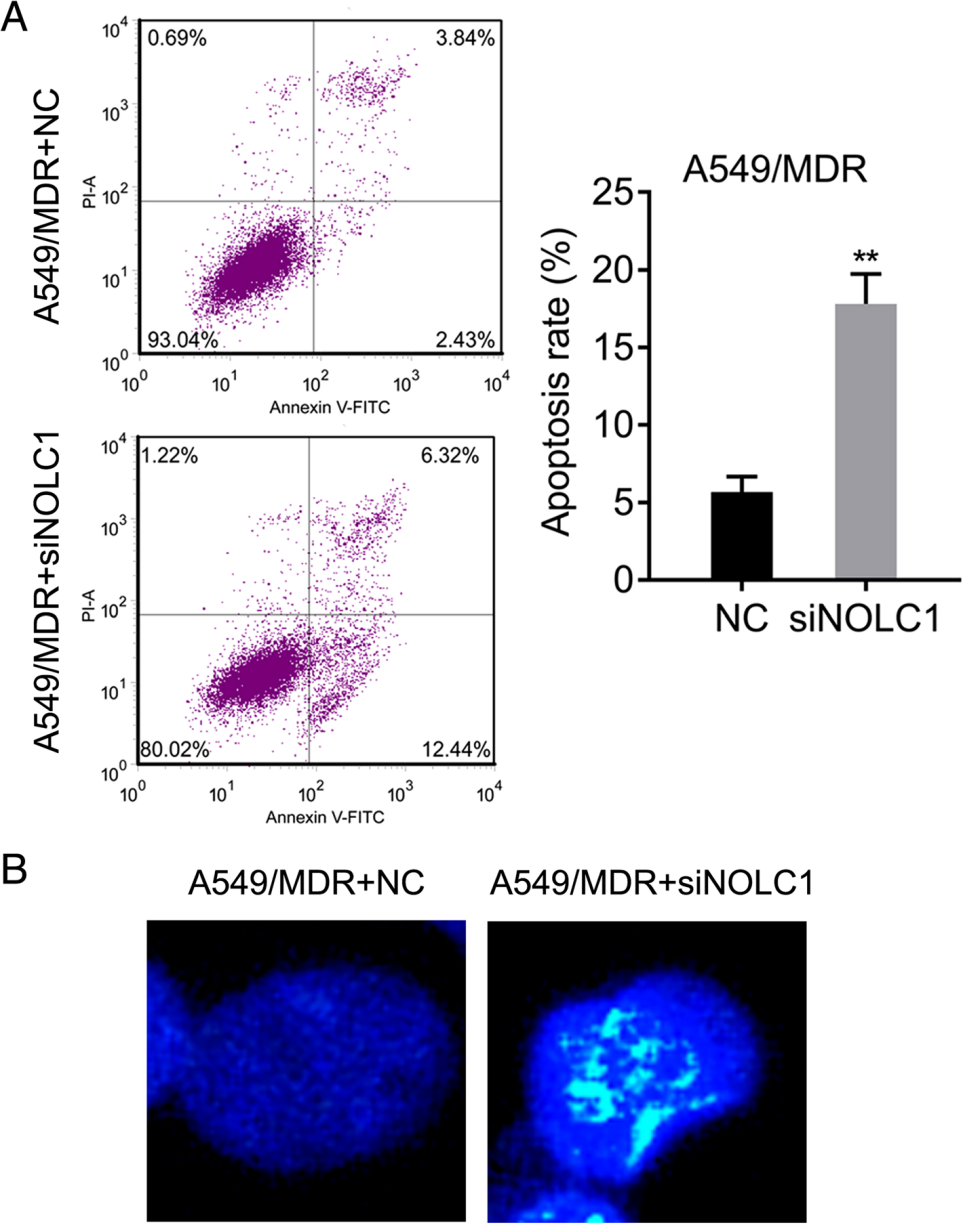
NOLC1 knockdown significantly promoted cell apoptosis in A549/MDR cells. **a** Flow cytometry analysis of A549/MDR cells stained with Annexin V and PI. The effect of siRNA-mediated NOLC1 knockdown on apoptosis was analyzed. Data were expressed as means ± SD of at least three experiments. **b** Hoechst staining assay was used to analyze the percentage of cells with fragmented nuclei in A549/MDR cells. ^**^p < 0.01 versus A549/MDR + NC

Based on the upregulated expression of NOLC1, we performed siRNA-mediated knockdown experiments to determine the effect of NOLC1 on chemosensitivity to multidrug in A549/MDR cell line. As shown in Fig. 4, knockdown of NOLC1 significantly enhanced the effects of 5-FU (*p* < 0.05), paclitaxel (*p* < 0.01), mitomycin (*p* < 0.05), DDP (*p* < 0.05) and gemcitabine hydrochloride (*p* < 0.05) on killing A549/MDR. Moreover, NOLC1 knockdown led to decreased IC50 and RI values under the treatments of 5-FU, paclitaxel, mitomycin, DDP and gemcitabine hydrochloride (Table 2). However, there was no significant difference in the effects of NOLC1 knockdown on killing A549/MDR after vinorelbine tartrate treatment. In addition, we evaluated whether knockdown of NOLC1 affected cell apoptosis of A549/MDR using flow cytometry and Hoechst 33258 staining. The results of flow cytometry indicated that the apoptotic rate of cells transfected with siNOLC1 or NC was 18.84 vs. 6.34% (*p* < 0.01) in A549/MDR cells (Fig. 5a). The results of Hoechst 33258 staining further confirmed that more cells with condensed and fragmented nuclei (indicating apoptotic nuclei) were presented in A549/MDR cells after NOLC1 knockdown compared with the control group (Fig. 5b). These data suggested that aberrant expression of NOLC1 may be responsible for acquired resistance to multidrug-based chemotherapeutic agents in NSCLC.

**Table 2:** Cytotoxicity of drugs in A549/MDR cell after siNOLC1 transfection

Drug	NC-IC50 (μg/ml)	siNOLC1-IC50 (μg/ml)	RI
5-fluorouracil	130.53	75.71	0.58
Paclitaxel	83.52	17.54	0.21
Mitomycin	8.64	3.89	0.45
Vinorelbine Tartrate	12.40	18.47	1.49
Cisplatin	33.00	10.89	0.33
Gemcitabine Hydrochloride	19.05	7.05	0.37

*IC50* 50% inhibitory concentration, *RI* resistance index, *DDP* Cisplatin, *MDR* multidrug resistance

### Knockdown of NOLC1 downregulated the expression of drug resistance-associated molecules in A549/MDR cells

**Fig. 6:**
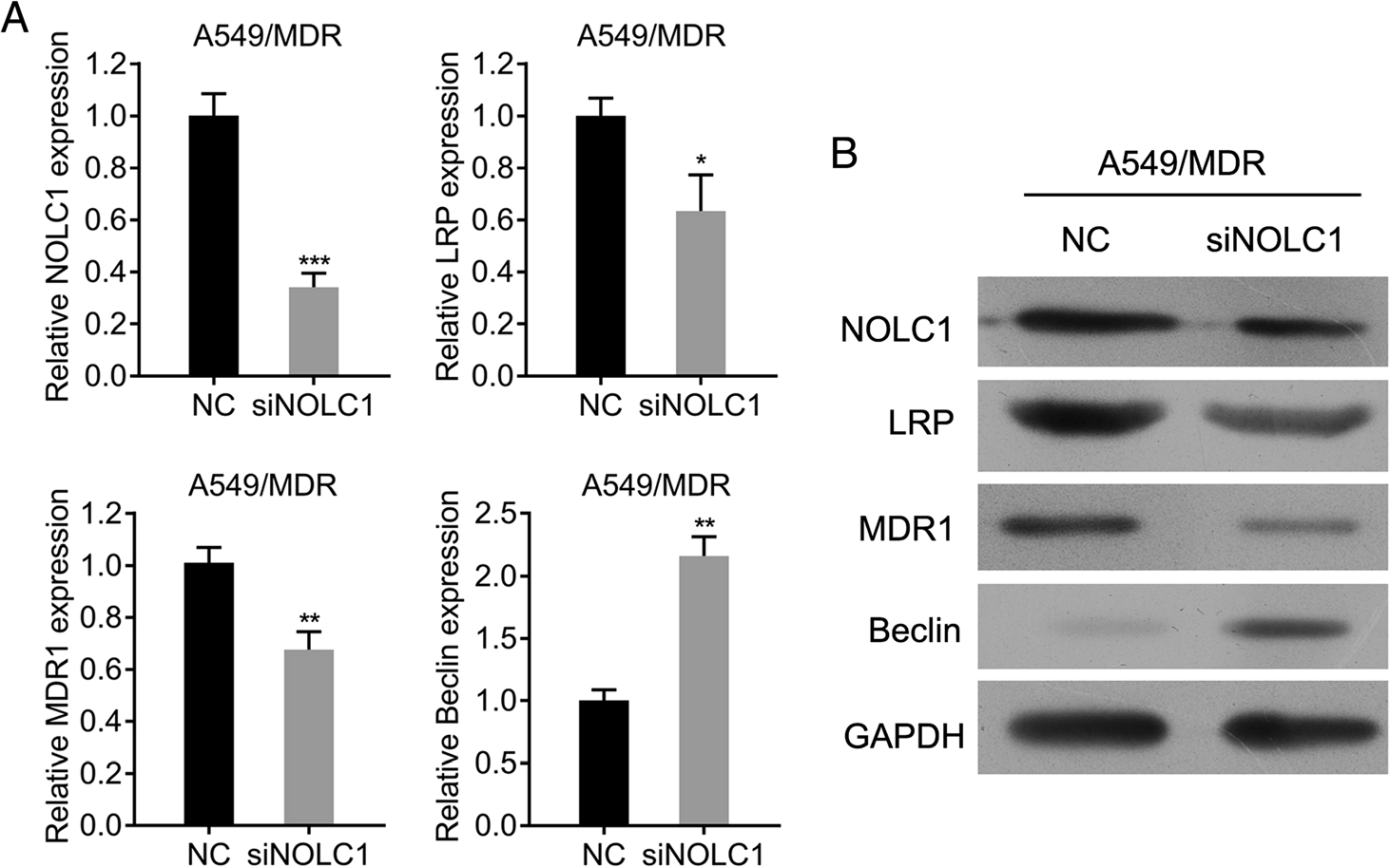
Effects of NOLC1 knockdown on drug resistance-associated expression in A549/MDR cells. The mRNA (**a**) and protein (**b**) expression of NOLC1, LRP, MRP and Beclin was determined by qRT-PCR and western blot analysis, respectively. GAPDH was used as internal controls. Data were expressed as means ± SD of at least three experiments. ^*^p < 0.05, ^**^p < 0.01, ^***^p < 0.001 versus A549/MDR + NC

To gain further insight into the mechanisms related to the effects of NOLC1 knockdown on killing A549/MDR, we investigated the effect of NOLC1 knockdown on the expression of LRP, MRP1 and Beclin using qRT-PCR and western blot analysis. As shown in Fig. 6a and b, siRNA against NOLC1 significantly decreased NOLC1 mRNA and protein expression levels in comparison with NC in A549/MDR (*p* < 0.001). Furthermore, NOLC1 knockdown significantly suppressed the expression of multidrug resistance (MDR1) (*p* < 0.01) and lung resistance related protein (LRP) (*p* < 0.05), but significantly elevated the expression of Beclin (*p* < 0.01).

## Discussion

NSCLC is one of the most lethal malignancies, with a poor 5-year survival rate. MDR developed in NSCLC has been a major obstacle in adjuvant chemotherapy [22, 23]. Even though molecular investigations have suggested several mechanisms underlying the development of MDR [24–26], the evidence on molecular alterations and pathway is far from sufficient.

In the present study, we constructed a multidrug resistant A549/MDR cell line of NSCLC from the A549/DDP cell line. Cytotoxicity testing proved that A549/MDR had acquired a stable multidrug resistance phenotype. Using microarray data, the gene expression pattern of these two cell lines was analyzed and a total of 921 DEGs were identified. According to GO and KEGG pathway annotation, these genes spanned many membranes of distinct functional families and biological processes and were involved in drug metabolism, chemical carcinogenesis, the Hippo signaling pathway, as well as transcriptional misregulation in cancer, which might play possible roles in MDR development in NSCLC therapy.

Expression levels of the mRNA of ten DEGs with the most significant differences were verified by qRT-PCR, which showed a good correlation with the microarray data. Among these verified DEGs, the NOLC1 expression difference was the most dramatic and thus it was selected for the in vitro experiments. NOLC1 (also known as NOPP140) was first identified as a nuclear localization signal-binding protein that also functions as a chaperone for shuttling between the nucleolus and cytoplasm [27, 28]. NOLC1 is also a novel nucleolar GTPase/ATPase and is reported to play a role in the regulation of tumorigenesis of lung cancer [29]. A previous study further demonstrated that NOLC1 is the target gene of p53 and it downregulation could be regulated by p53 upregulation involved in molecular pathways of tumor suppressor genes [30]. Alterations of nucleolar phosphoprotein p130 encoded by the NOLC1 gene during mitosis are correlated well with the nucleolar disassembly and reassembly [31]. Moreover, a highly phosphorylated human p130 was found to be expressed in synchrony with cell-growth activation and cell cycle dependent alterations [32, 33]. In nasopharyngeal carcinoma, NOLC1 and tumor protein 53 synergistically co-regulated a cellular proto-oncogene MDM2, leading to cell growth and reduction of apoptosis [34]. Consistent with these facts, the in vitro experiments indicated that knockdown of NOLC1 enhanced the drug sensitivity of A549/MDR cells in response to multidrug by suppressing cell viability and promoting apoptosis. Notably, we found that NOLC1 knockdown caused a significant RI decrease in response to multidrug, but a slight increase after vinorelbine tartrate treatment in A549/MDR, which might be ascribed to the property of vinorelbine tartrate, cell types or the endogenous signaling pathways regulated by NOLC1. Furthermore, we found that the expression of MDR1 and LRP was decreased, but Beclin was increased at mRNA and protein levels in A549/MDR cells after NOLC1 knockdown. MDR1 encodes p-glycoprotein (P-gp) and was first reported to influence MDR development by Kornmann et al. [35]. LRP has been shown to be increased in the resistance of A549 cells to Dox treatment via its accumulation in intracytoplasmic vesicles [36]. Beclin, an autophagy-related gene, has been reported to be associated with cell apoptosis and autophagy in anti-tumor therapy [37].

In conclusion, the present study used DNA microarray analysis to provide a new and comprehensive expression profile of MDR in NSCLC cells. The selected NOLC1 gene might play an important role in regulating chemosensitivity of NSCLC cells by promoting apoptosis and regulating drug resistance-associated molecules. However, the biological function and mechanism of these DEGs and the target genes in NSCLC chemoresistance still need further experimental exploration, which will provide new insights in understanding the molecular mechanisms of MDR.

## Additional files

Additional file 1: **Table S1.** Primers for quantitative real-time polymerase chain reaction (qRT-PCR). (DOCX 15 kb)

Additional file 2: **Table S2.** Top 40 differentially expressed genes between A549/MDR cells and A549/DDP cells. (DOCX 16 kb)

Additional file 3: **Table S3.** Classification of upregulated DEGs between A549/MDR cells and A549/DDP cells according to GO terms with *p* value < 0.05. DEGs: differentially expressed genes; GO, Gene Ontology. (DOCX 15 kb)

Additional file 4: **Table S4.** Classification of downregulated DEGs between A549/MDR cells and A549/DDP cells according to GO terms with *p* value < 0.05. DEGs: differentially expressed genes; GO, Gene Ontology. (DOCX 15 kb)

## Abbreviations

5-FU: 5-fluorouracil
ABC: ATP-binding cassette
BCA: Bicinchoninic acid
CCK-8: Cell Counting Kit-8
DDP: Cisplatin
DEGs: Differentially expressed genes
DMEM: Dulbecco’s modified Eagle medium
FBS: Fetal bovine serum
MDR: Multidrug resistance
MRP: Multidrug resistance-associated protein
NC: Negative control
NSCLC: Non-small cell lung cancer
qRT-PCR: Quantitative real-time PCR
RI: Resistance index
RIPA: Radioimmunoprecipitation assay
