# Supplementary material for: Identification and validation of NOLC1 as a potential target for enhancing sensitivity in multidrug resistant non-small cell lung cancer cells
Source: Cell Mol Biol Lett. 2018 Nov 27;23:54. doi: 10.1186/s11658-018-0119-8 (PMC6258490; doi:10.1186/s11658-018-0119-8)
Supplement: Supplementary file 1 — Table S1. Primers for quantitative real-time polymerase chain reaction (qRT-PCR). (DOCX 15 kb) [file 11658_2018_119_MOESM1_ESM.docx]

**Table S1.** Primers for quantitative real-time polymerase chain reaction (qRT-PCR).

| **Gene** | **Forward (5′ - 3′)** | **Reverse (5′ - 3′)** |
| --- | --- | --- |
| CASQ2 | CAAACTGGAAGTCCAAGCCTT | GGCTCATCCATAAATGGCTCAT |
| CEMP1 | TCTTTGGACCTGGAGACATTTC | GCTTCTGGAGCCCTCTTGG |
| EMP2 | ATTCACGACAAAAACGCGAAAT | CAGTATCAGGTACATCATGCCG |
| NOLC1 | AAGAAGCCACAGAAGGTAGCA | CACTGGAGTCATCAGAAGAAGAAC |
| SLC24A3 | GGAAACAGTGAAATGGGCGT | AGCAAACGTCACCATGAACC |
| ANP32E | AGGAGGTGACAGAGTTAGTCC | ACATTTCTCTGCCAGGACTTC |
| ARAP1 | AGGATGACCACGCCTATGAG | CCTGCGGTGGGTTCTTGTC |
| CXCR3 | ATGGAGTTGAGGAAGTACGG | CCTGGCAGGAACTCTTTTGT |
| IL36G | AGTTCCACGAAGTGACAGTG | CAGTCTTGGCACGGTAGAAA |
| ST5 | CTGCCACGATTACCCAAGAGG | AGGCTGTCTTCATTGAGGCTG |
| LRP | GGCACTTTGAGGTGAATGAC | GCGGGCTGAGTTCTTATGG |
| MDR1 | GGGATGGTCAGTGTTGATGGA | GCTATCGTGGTGGCAAACAATA |
| Beclin-1 | CCATGCAGGTGAGCTTCGT | GAATCTGCGAGAGACACCATC |
| GAPDH | TGTTCGTCATGGGTGTGAAC | ATGGCATGGACTGTGGTCAT |
