# Supplementary material for: Identification and validation of NOLC1 as a potential target for enhancing sensitivity in multidrug resistant non-small cell lung cancer cells
Source: Cell Mol Biol Lett. 2018 Nov 27;23:54. doi: 10.1186/s11658-018-0119-8 (PMC6258490; doi:10.1186/s11658-018-0119-8)
Supplement: Supplementary file 2 — Table S2. Top 40 differentially expressed genes between A549/MDR cells and A549/DDP cells. (DOCX 16 kb) [file 11658_2018_119_MOESM2_ESM.docx]

**Table S2.** Top 40 differentially expressed genes between A549/MDR cells and A549/DDP cells.

| **Gene** | **Regulation** | **Fold Change** | **p-value** |
| --- | --- | --- | --- |
| EMP2 | up | 2.48 | 0.022 |
| SLC24A3 | up | 2.44 | 0.015 |
| CASQ2 | up | 2.33 | 0.033 |
| NOLC1 | up | 2.28 | 0.026 |
| CEMP1 | up | 2.43 | 0.025 |
| CHRNA6 | up | 1.78 | 0.015 |
| ACKR4 | up | 1.53 | 0.011 |
| FSHB | up | 2.10 | 0.023 |
| CTNNBIP1 | up | 1.69 | 0.042 |
| STMN4 | up | 1.76 | 0.023 |
| PTPRR | up | 1.50 | 0.012 |
| CD36 | up | 1.66 | 0.023 |
| IL1RAPL1 | up | 1.74 | 0.028 |
| CREB5 | up | 1.92 | 0.046 |
| GSTM2 | up | 1.51 | 0.035 |
| ARL14 | up | 1.87 | 0.014 |
| SPTBN1 | up | 1.59 | 0.023 |
| CAMK1D | up | 1.77 | 0.024 |
| TMC8 | up | 1.57 | 0.022 |
| HMGCS2 | up | 2.30 | 0.043 |
| ST5 | down | 0.34 | 0.002 |
| BEND3 | down | 0.40 | 0.044 |
| ARAP1 | down | 0.37 | 0.003 |
| CXCR3 | down | 0.40 | 0.005 |
| ANP32E | down | 0.41 | 0.011 |
| IL36G | down | 0.40 | 0.013 |
| POMZP3 | down | 0.41 | 0.032 |
| OSBP2 | down | 0.44 | 0.014 |
| STOML1 | down | 0.40 | 0.016 |
| GPRC5A | down | 0.42 | 0.017 |
| CHRFAM7A | down | 0.44 | 0.009 |
| FAM205BP | down | 0.44 | 0.015 |
| THEM4 | down | 0.45 | 0.018 |
| METTL21C | down | 0.47 | 0.014 |
| DST | down | 0.39 | 0.017 |
| IRGM | down | 0.41 | 0.015 |
| TRIM45 | down | 0.48 | 0.024 |
| ADAM11 | down | 0.48 | 0.031 |
| NOL4 | down | 0.40 | 0.034 |
| CNIH2 | down | 0.43 | 0.041 |
