# Supplementary material for: Identification and validation of NOLC1 as a potential target for enhancing sensitivity in multidrug resistant non-small cell lung cancer cells
Source: Cell Mol Biol Lett. 2018 Nov 27;23:54. doi: 10.1186/s11658-018-0119-8 (PMC6258490; doi:10.1186/s11658-018-0119-8)
Supplement: Supplementary file 3 — Table S3. Classification of upregulated DEGs between A549/MDR cells and A549/DDP cells according to GO terms with p value < 0.05. DEGs: differentially expressed genes; GO, Gene Ontology. (DOCX 15 kb) [file 11658_2018_119_MOESM3_ESM.docx]

**Table S3.** Classification of upregulated DEGs between A549/MDR cells and A549/DDP cells according to GO terms with *p* value < 0.05.

| **GO terms** | **Count** | **P-value** |
| --- | --- | --- |
| **Biological process (BP)** |  |  |
| GO:0090150, establishment of protein localization to membrane | 22 | 0.015 |
| GO:0006898, receptor-mediated endocytosis | 17 | 0.015 |
| GO:1902578, single-organism localization | 27 | 0.015 |
| GO:1902580, single-organism cellular localization | 27 | 0.015 |
| GO:0060047, heart contraction | 15 | 0.016 |
| GO:0003015, heart process | 15 | 0.016 |
| GO:0072657, protein localization to membrane | 24 | 0.016 |
| GO:0031175, neuron projection development | 38 | 0.036 |
| GO:0006936, muscle contraction | 19 | 0.047 |
| GO:0051129, negative regulation of cellular component organization | 26 | 0.047 |
| **Cellular component (CC)** |  |  |
| GO:0030529, ribonucleoprotein complex | 38 | 4.11E-05 |
| GO:0005840, ribosome | 20 | 4.11E-05 |
| GO:0005737, cytoplasm | 280 | 4.68E-05 |
| GO:0044424, intracellular part | 341 | 3.49E-04 |
| GO:0005622, intracellular | 343 | 3.72E-04 |
| GO:0044422, organelle part | 205 | 4.84E-04 |
| GO:0043226, organelle | 327 | 4.84E-04 |
| GO:0044444, cytoplasmic part | 214 | 4.99E-04 |
| GO:0032991, macromolecular complex | 145 | 5.45E-04 |
| GO:0044446, intracellular organelle part | 198 | 9.29E-04 |
| **Molecular function (MF)** |  |  |
| GO:0005198, structural molecule activity | 42 | 1.31E-06 |
| GO:0044822, poly(A) RNA binding | 54 | 6.35E-04 |
| GO:0043295, glutathione binding | 5 | 8.57E-04 |
| GO:1900750, oligopeptide binding | 5 | 8.57E-04 |
| GO:0038024, cargo receptor activity | 9 | 3.50E-03 |
| GO:1901681, sulfur compound binding | 16 | 6.52E-03 |
| GO:0003723, RNA binding | 61 | 7.70E-03 |
| GO:0005515, protein binding | 240 | 7.70E-03 |
| GO:0003735, structural constituent of ribosome | 13 | 8.39E-03 |
| GO:0044548, S100 protein binding | 4 | 0.010 |

DEGs: differentially expressed genes; GO, Gene Ontology
