# Supplementary material for: Identification and validation of NOLC1 as a potential target for enhancing sensitivity in multidrug resistant non-small cell lung cancer cells
Source: Cell Mol Biol Lett. 2018 Nov 27;23:54. doi: 10.1186/s11658-018-0119-8 (PMC6258490; doi:10.1186/s11658-018-0119-8)
Supplement: Supplementary file 4 — Table S4. Classification of downregulated DEGs between A549/MDR cells and A549/DDP cells according to GO terms with p value < 0.05. DEGs: differentially expressed genes; GO, Gene Ontology. (DOCX 15 kb) [file 11658_2018_119_MOESM4_ESM.docx]

**Table S4.** Classification of downregulated DEGs between A549/MDR cells and A549/DDP cells according to GO terms with *p* value < 0.05.

| **GO terms** | **Count** | **P-value** |
| --- | --- | --- |
| **Biological process (BP)** |  |  |
| GO:0009953, dorsal/ventral pattern formation | 8 | 2.93E-04 |
| GO:0031061, negative regulation of histone methylation | 3 | 9.61E-04 |
| GO:0031057, negative regulation of histone modification | 4 | 0.001 |
| GO:1903309, negative regulation of chromatin modification | 4 | 0.002 |
| GO:0060348, bone development | 8 | 0.002 |
| GO:0031128, developmental induction | 4 | 0.002 |
| GO:0045168, cell-cell signaling involved in cell fate commitment | 4 | 0.002 |
| GO:0035136, forelimb morphogenesis | 4 | 0.003 |
| GO:0035590, purinergic nucleotide receptor signaling pathway | 3 | 0.003 |
| GO:0003002, regionalization | 13 | 0.003 |
| **Cellular component (CC)** |  |  |
| GO:0031463, Cul3-RING ubiquitin ligase complex | 3 | 0.006 |
| GO:0030130, clathrin coat of trans-Golgi network vesicle | 2 | 0.012 |
| GO:0012510, trans-Golgi network transport vesicle membrane | 2 | 0.015 |
| GO:0005923, tight junction | 5 | 0.024 |
| GO:0070160, occluding junction | 5 | 0.024 |
| GO:0030665, clathrin-coated vesicle membrane | 4 | 0.028 |
| GO:0005776, autophagic vacuole | 3 | 0.028 |
| GO:0001673, male germ cell nucleus | 2 | 0.032 |
| GO:0030125, clathrin vesicle coat | 2 | 0.035 |
| GO:0030658, transport vesicle membrane | 4 | 0.040 |
| **Molecular function (MF)** |  |  |
| GO:0003730, mRNA 3′-UTR binding | 4 | 0.002 |
| GO:0001614, purinergic nucleotide receptor activity | 3 | 0.003 |
| GO:0016502, nucleotide receptor activity | 3 | 0.003 |
| GO:0035586, purinergic receptor activity | 3 | 0.005 |
| GO:0030145, manganese ion binding | 4 | 0.006 |
| GO:0005501, retinoid binding | 3 | 0.012 |
| GO:0019840, isoprenoid binding | 3 | 0.012 |
| GO:0001608, G-protein coupled nucleotide receptor activity | 2 | 0.013 |
| GO:0045028, G-protein coupled purinergic nucleotide receptor activity | 2 | 0.013 |
| GO:0008134, transcription factor binding | 14 | 0.013 |

DEGs: differentially expressed genes; GO, Gene Ontology
